# Renin Promotes STAT4 Phosphorylation to Induce IL-17 Production in Keratinocytes of Oral Lichen Planus

**DOI:** 10.1016/j.isci.2020.100983

**Published:** 2020-03-13

**Authors:** Xuejun Ge, Hanting Xie, Tivoli Nguyen, Bin Zhao, Jing Xu, Jie Du

**Affiliations:** 1 Department of Endodontics, Shanxi Medical University School and Hospital of Stomatology, Taiyuan, Shanxi, China; 2 Department of Pathology, Shanxi Medical University, Taiyuan, Shanxi, China; 3 Division of Biological Sciences, Department of Medicine, The University of Chicago, Chicago, IL, USA; 4 Department of Oral Medicine, Shanxi Medical University School and Hospital of Stomatology, NO. 56 Xinjian South Road, Taiyuan, Shanxi 030001, China; 5 Department of Prosthodontics, Shanxi Medical University School and Hospital of Stomatology, Taiyuan, Shanxi, China; 6 Shanxi Province Key Laboratory of Oral Diseases Prevention and New Materials, Taiyuan, Shanxi, China

**Keywords:** Oral Medicine, Molecular Biology, Immunology

## Abstract

Interleukin-17 (IL-17) is highly expressed in the epithelial layer of oral lichen planus (OLP), but the underlying mechanism for IL-17 overexpression remains unknown. Here, we identify renin that is induced by NF-κB pathway contributes to the increase of IL-17 in human oral keratinocytes (HOKs). We describe that the release of cellular renin leads to the phosphorylation of Janus kinase 2 (JAK2) protein. The phosphorylated JAK2 recruits and activates the signal transducer and activator of transcription 4 (STAT4) by phosphorylating STAT4's tyrosine residue 693 (Tyr693). The now-activated STAT4 translocates into nucleus and binds to the promoter region of *IL-17* gene in HOKs. Genetic interference of renin restores IL-17 levels in OLP cell models. Collectively, our results reveal that renin upregulates IL-17 expression by enhancing STAT4 phosphorylation. This discovery unveils an underpinning by which IL-17 is increased in oral keratinocytes and provides potential targeted therapies for OLP patients.

## Highlights

• NF-κB pathway induces renin expression in oral lichen planus
• Renin upregulation promotes IL-17 expression in oral keratinocytes
• Renin regulates IL-17 by promoting STAT4 phosphorylation in oral keratinocytes

## Graphical Abstract

**Figure:**
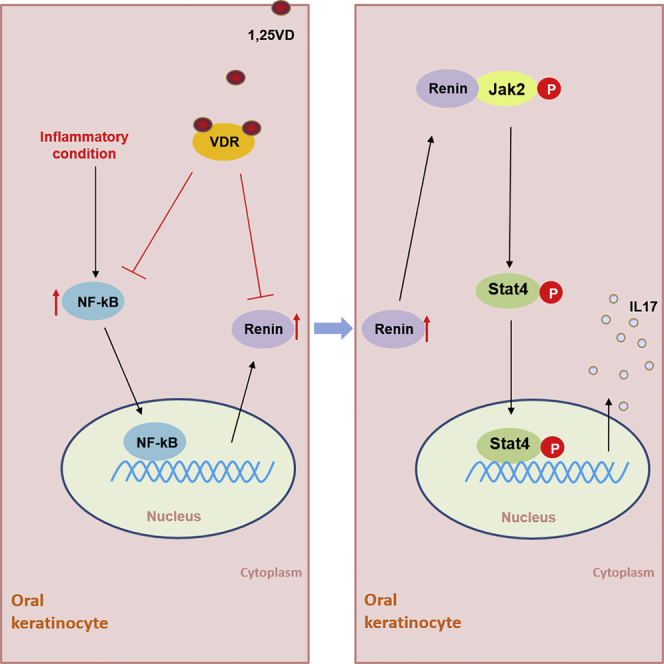

## Introduction

Oral lichen planus (OLP) is a chronic relapsing inflammatory disorder affecting the mucous membranes of gums, buccal mucosa, palate, and tongue(Cheng et al., 2016). Crucial in the initiation and development of this disease is T-lymphocyte infiltration, an event that triggers inflammatory responses of both the lamina propria and the epithelial layer and leads to keratinocyte apoptosis in oral epithelium (Lavanya et al., 2011). Although numerous studies have suggested that OLP is an autoimmune response-driven disease in a common consensus, the exact pathogenesis remains unclear. To date, multiple pathogenic factors, such as autoimmune response, mental stress, infection, and hypersensitivity, have been found to contribute to OLP onset (Cheng et al., 2016). There are six recognized subtypes of clinical expression for OLP: erosive, plaque, papular, reticular, atrophic, and bullous (Cheng et al., 2016, Gorouhi et al., 2014). Patients who are smokers and alcoholics take more risks for malignant transformation (Aghbari et al., 2017). Because OLP is refractory in clinic, much attention has been given to symptomatic improvement (Cheng et al., 2016). At present, topical and systemic immunosuppressants are typically administered to relieve clinical symptoms, but these leave significant side effects upon long-term management (Cheng et al., 2016, Crincoli et al., 2011, De Rossi and Ciarrocca, 2014). Thus, explorations regarding the pathogenies of OLP and curative treatments of it are required.

T-helper (Th) 17, a CD4 T cell subset, has been described to play a prominent role in allergic reactions, autoimmune development, and host defense by generating IL-17, a type of pro-inflammatory cytokine (Bettelli et al., 2006). IL-17 can activate diverse cells such as epithelial cell, fibroblast, and chondrocyte to produce powerful inflammatory molecules (Gaffen, 2008, Onishi and Gaffen, 2010). Exogenous IL-17 in oral keratinocytes has been reported to induce CCL-20, IL-8, and TNF-α production (Lu et al., 2014). Previous studies have stated that, compared with unaffected controls, increased levels of IL-17 mRNA and protein are detected in OLP (Lu et al., 2014), indicating IL-17 may have a critical role in OLP disease. Interestingly, serum IL-17 concentrations in erosive OLP patients are higher than those in the non-erosive subtype (Pouralibaba et al., 2013), suggesting a positive correlation between IL-17 levels and OLP severity.

Renin is a key component of the renin-angiotensin system (RAS), which is broadly recognized to mediate blood pressure and vascular resistance (Re, 2004). After being synthesized in kidney, renin is secreted into the circulation in which angiotensinogen is cleaved to angiotensin I, which is converted to angiotensin II further by angiotensin-converting enzyme to exert biological and physiological functions (He et al., 2019, Sparks et al., 2014). In addition to playing a known role in circulatory regulation, RAS has been emphasized to result in autoimmune disorders recently by some studies that suggest RAS facilitates colitis through activating Th17 cells, and renin expression is significantly increased in inflamed colonic tissues (He et al., 2019). Moreover, RAS suppression limits IL-17 production in lung diseases (Weber et al., 2012), implying renin may be involved in regulating IL-17 expression. Given the important roles of IL-17 in OLP and the relationship between RAS and IL-17, in this study, we explored renin as well as IL-17 in the context of OLP and found that the overexpression of renin enhances IL-17 production by phosphorylating signal transducer of activated transcription 4 (STAT4) in oral keratinocytes of OLP.

## Results

### Renin Levels Are Upregulated in the Field of OLP

**Figure 1:**
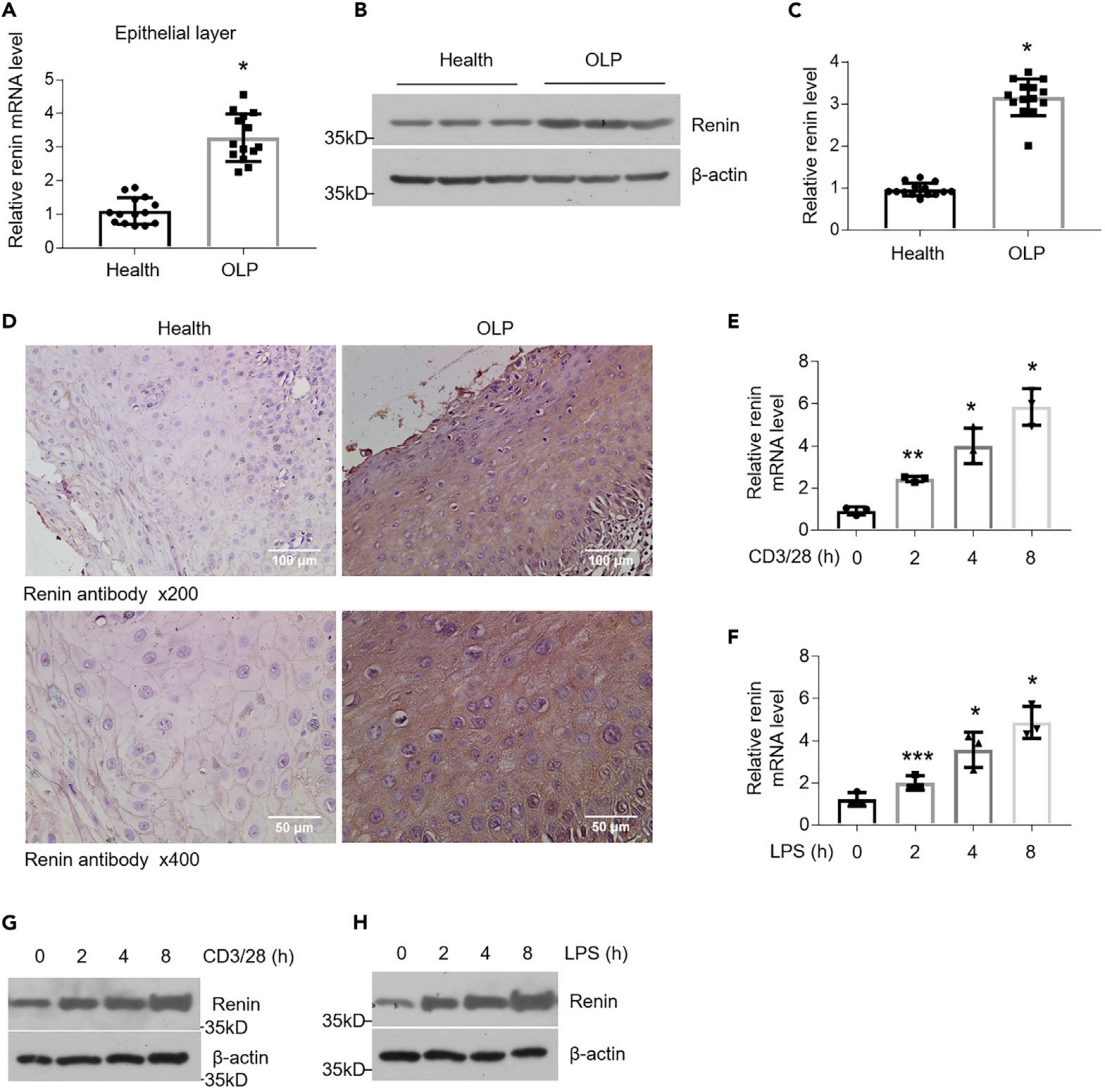
Renin Expression Is Upregulated in the Keratinocytes of OLP (A) Real-time PCR quantification of renin in the oral mucosal epithelial layer, n = 14 each group. (B and C) Western blot analysis (B) and densitometric quantitation (C) of renin in epithelial layer of oral mucosa, n = 14 each group. (D) Renin immunostaining in the oral tissues of healthy controls and OLP patients. (E and F) Real-time PCR quantification of renin in HOKs challenged by anti-CD3/28-activated CD4^+^ T cells (E) or 100 ng/mL LPS (F) for 0, 2, 4, 8 h, n = 3. (G and H) Western blot determination of renin in HOKs challenged by anti-CD3/28-activated CD4^+^ T cells (G) or 100 ng/mL LPS (H) for different time points as shown, n = 3. ∗p < 0.05, ∗∗p < 0.01, ∗∗∗p < 0.001 versus corresponding control. Data were shown as means ± SD. Two-tailed Student's *t* test was used.

Because renin is reported to be involved in autoimmune diseases (He et al., 2019), we collected oral biopsies from patients with OLP and healthy individuals, sampled from clinically lesion and unaffected mucosa, to test renin expression in these tissues. As shown in Figure 1, mRNA and protein levels of renin showed increases of more than 3-fold in the epithelial layer of specimens derived from OLP in comparison to healthy controls (Figures 1A–1C). Our immunohistochemistry staining data confirmed the upregulation of renin in OLP and indicated that the overexpressed renin is localized in the cytoplasm of oral keratinocyte rather than in nucleus (Figure 1D). Furthermore, the enhanced expression of renin in the lamina propria of OLP was detected as well (Figures S1A and S1B). To gain a better understanding of the molecular mechanism of OLP development, we employed LPS and the culture medium of activated CD4^+^ T cells to establish OLP cell models as described in previous investigation (Zhao et al., 2019). Consistent with our hypothesis, renin mRNA and protein levels were elevated by LPS or activated CD4^+^ T cell stimulation in a time-dependent manner (Figures 1E–1H). These results demonstrate that renin is induced under OLP circumstances.

### The Increase of Renin Is Regulated by NF-κB Pathway in Oral Keratinocytes

**Figure 2:**
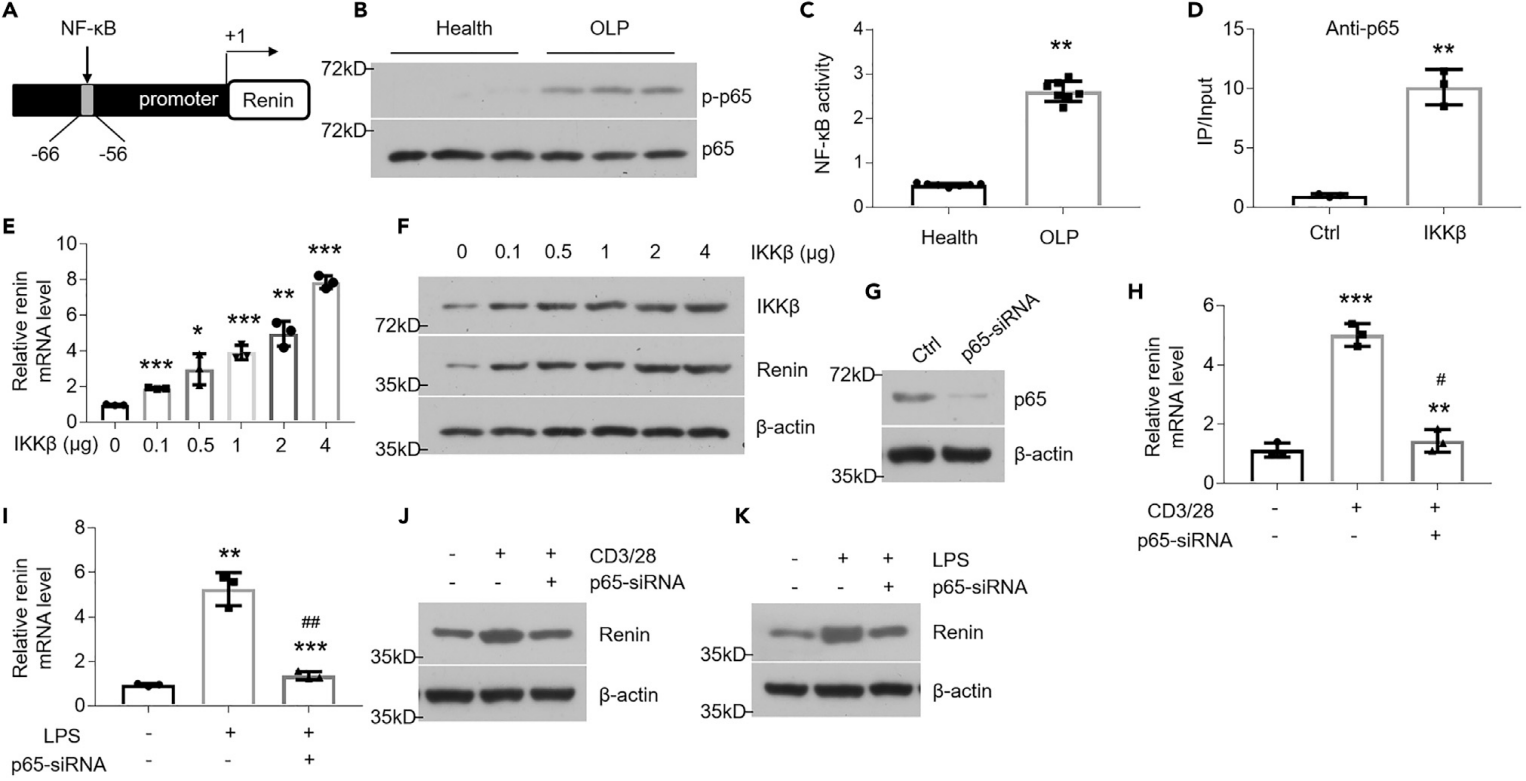
Renin Is Induced by NF-κBPathway (A) Schematic illustration of κB motif in the promoter of *renin* gene. (B) Western blot analysis of phosphorylated NF-κB p65 in the epithelial layer of human samples, n = 14. (C) NF-κB activity in the epithelial layer of human samples, n = 7. (D) IKKβ plasmids transfection in HOKs helps NF-κB bind to the promoter of endogenous *renin*. Chromatin immunoprecipitation (ChIP) was performed using anti-p65 antibody. (E and F) Real-time PCR (E) and Western blot (F) analyses of renin in HOKs transfected with IKKβ plasmids. (G) Efficacy of siRNA-mediated knockdown on p65 expression was detected by Western blot in HOKs. (H–K) Quantitative PCR (H and I) and Western blot (J and K) analyses of renin levels in HOKs, which were treated with activated CD4^+^ T cells or 100 ng/mL LPS for 8 h, following 36-h scramble- or p65-siRNA transfection. ∗p < 0.05, ∗∗p < 0.01, ∗∗∗p < 0.001 versus corresponding control; #p < 0.05, ##p < 0.01 versus LPS or CD3/28 group, n = 3. Ctrl, control. Data were shown as means ± SD. Two-tailed Student's *t* test and one-way analysis of variance were used.

The mechanism of renin's upregulation under inflammatory condition is still not well explained. To explore this, we examined human *renin* gene promoter and found a putative κB motif (Figure 2A). The NF-κB pathway, which plays a critical mediatory role in OLP, was found to be robustly activated in diseased tissues and cell models (Figures 2B, 2C, S2A, and S2B). We next designed primers flanking the κB-binding site and performed ChIP assays in HOKs transfected with IKKβ plasmid reportedly being an activator for NF-κB pathway (Chen et al., 2013). As shown in Figure 2, the binding of p65 to the κB site was accelerated by IKKβ plasmid transfection (Figure 2D), stating NF-κB pathway most likely regulates renin transcription. Next, we performed ChIP assays in both human biopsies and OLP cell models and these results coincide with previous findings (Figures S2C–S2E). To determine whether the activated NF-κB pathway strengthens renin expression in HOKs, we transfected IKKβ plasmids into this cell line and found that renin levels were increased in the presence of IKKβ overexpression in a dose dependent-manner (Figures 2E and 2F). In the loss-of-function assays, we silenced the p65 gene using siRNAs in order to suppress NF-κB pathway as described previously (Wu et al., 2017) (Figure 2G). As shown, inhibition of NF-κB pathway blocked the enhanced renin expression induced by LPS or activated CD4^+^ T cells (Figures 2H–2K). Bay 11-7082 compound, an NF-κB pathway inhibitor, also decreased renin levels in OLP cell models (Figures S2F and S2G).

### Renin Mediates IL-17 Expression in HOKs

**Figure 3:**
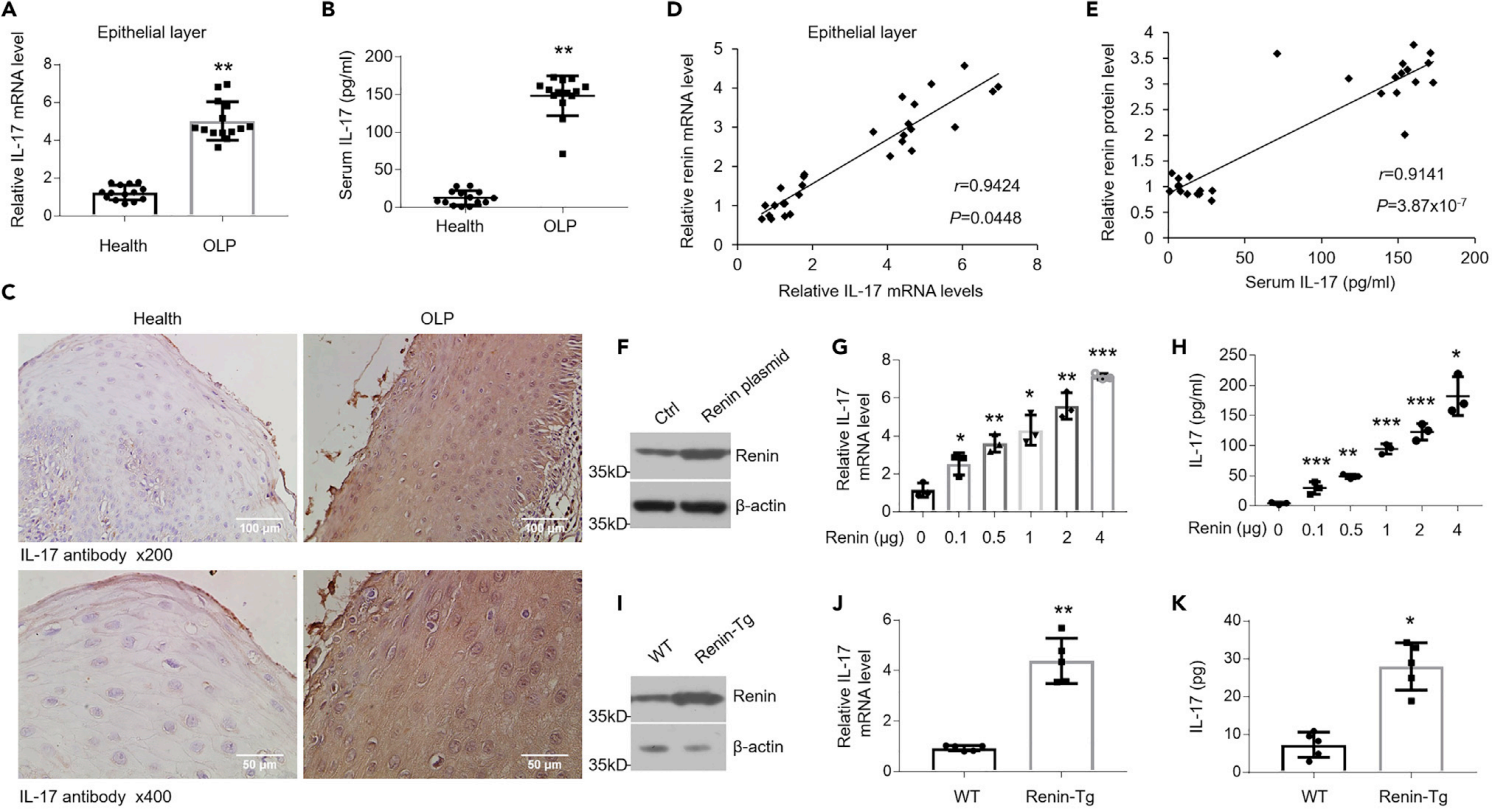
Renin Upregulates IL-17 Expression in Oral Keratinocytes (A) Real-time PCR quantification of IL-17 mRNA levels in oral keratinocytes from human specimens, n = 14. (B) ELISA measurement of IL-17 concentrations in serum from healthy individuals or OLP patients, n = 14. (C) IL-17 immunostaining in the oral tissues of healthy controls and OLP patients. (D) Correlation of fold change between renin mRNA levels and IL-17 mRNA status in oral epitheliums of human biopsies. (E) Correlation between renin protein levels in the human oral epithelial layer and IL-17 concentrations in serum of participants. (F) Western blot analysis of renin protein levels in HOKs transfected with empty or renin plasmids, n = 3. (G) Real-time PCR quantification of IL-17 mRNA levels in HOKs after renin transfection with different doses as shown, n = 3. (H) ELISA detection of IL-17 productions in the culture medium of HOKs after renin transfection with different doses as shown, n = 3. (I and J) Western blot (I) of renin expression or quantitative PCR test (J) of IL-17 mRNA levels in oral keratinocytes obtained from wild-type or renin-transgene mice, n = 5. (K) ELISA examination of IL-17 expression in oral keratinocytes obtained from wild-type or renin-transgene mice, n = 5. ∗p < 0.05, ∗∗p < 0.01, ∗∗∗p < 0.001 versus corresponding control. Ctrl, control; WT, wild type. Data were shown as means ± SD. Two-tailed Student's *t* test was used.

Some studies have reported renin to be closely related with IL-17 expression (He et al., 2019). To explore this, we first examined IL-17 status in the context of OLP. As expected, compared with healthy controls, mRNA and protein levels of IL-17 in oral epithelia and secretion of IL-17 in serum were increased for OLP patients (Figures 3A–3C). Renin and IL-17 levels of participants showed good positive correlations (Figures 3D and 3E). Accordantly, IL-17 expression was elevated in the OLP cell models in a time-course-dependent manner (Figures S3A–S3D). Does renin regulate IL-17 expression in HOKs? To answer this question, we transfected human renin plasmids into HOKs (Figure 3F). As manifested, IL-17 was induced in a dose-dependent fashion after transfection (Figures 3G and 3H). IL-17 expression was also enhanced after recombinant renin treatment in HOKs (Figure S3E). In agreement, the expression of IL-17 in oral epithelia from renin transgene mice was considerably increased (Figures 3I–3K). IKKβ overexpression was also shown to stimulate IL-17 transcription and production in a dose-dependent way (Figures S3F and S3G).

**Figure 4:**
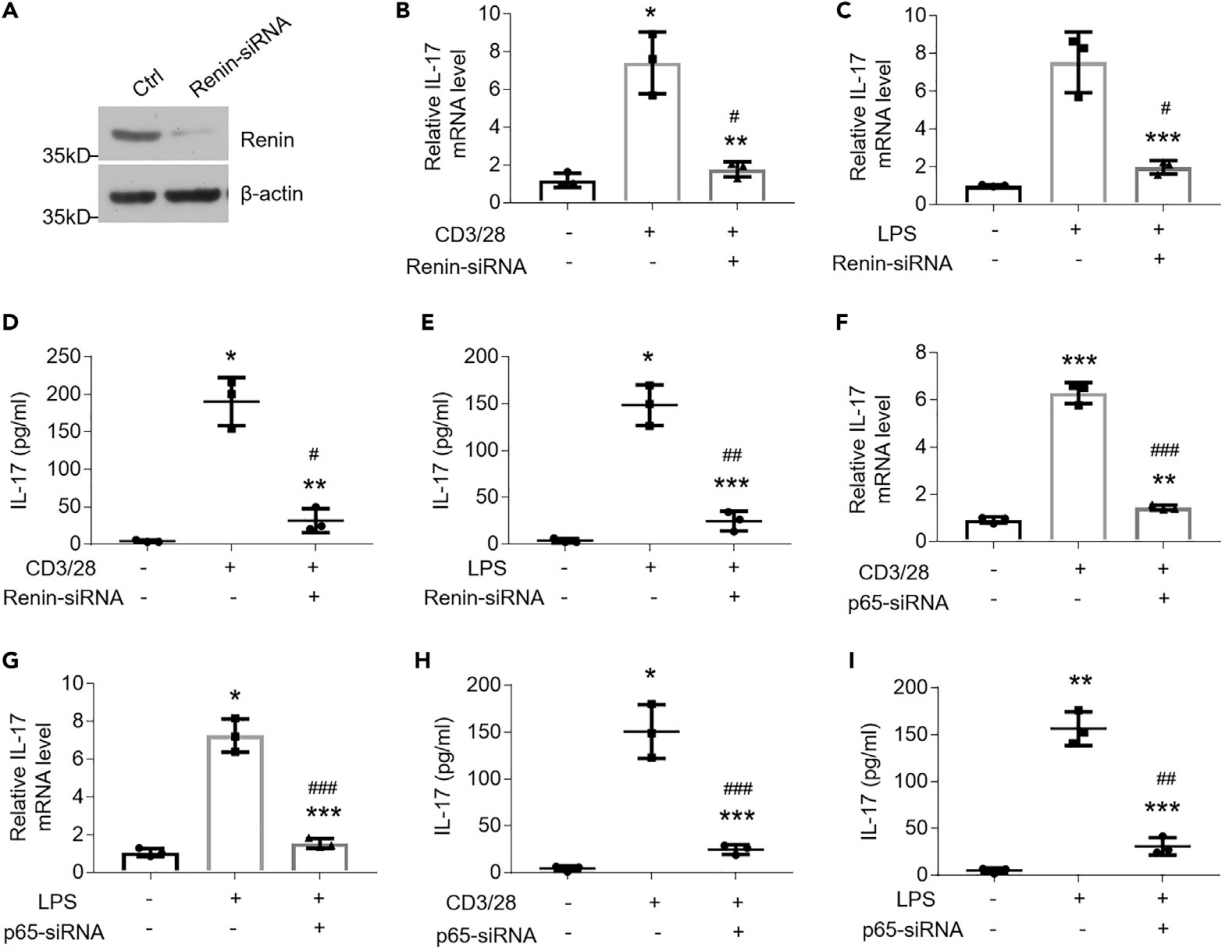
Interference of Renin Prohibits IL-17 Productions in OLP Cell Models (A) Efficacy of siRNA-mediated knockdown on renin expression was detected by Western blot in HOKs. (B and C) Real-time PCR quantification of IL-17 mRNA levels in HOKs stimulated with activated CD4^+^ T cells (B) or 100 ng/mL LPS (C) for 8 h, following 36-h scramble- or renin-siRNA pretreatment. (D and E) ELISA detection of IL-17 secretions in the culture medium from HOKs stimulated with activated CD4^+^ T cells (D) or 100 ng/mL LPS (E) for 8 h, following 36-h scramble- or renin-siRNA pretreatment. (F–I) HOKs were transfected with scramble- or p65-siRNA for 36 h, followed by 8-h activated CD4^+^ T cells or 100 ng/mL LPS treatment. Real-time PCR (F and G) and ELISA (H and I) tests were performed. ∗p < 0.05, ∗∗p < 0.01, ∗∗∗p < 0.001 vs. corresponding control; #p < 0.05, ##p < 0.01, ###p < 0.001 versus LPS or CD3/28 group, n = 3. Ctrl, control. Data were shown as means ± SD. Two-tailed Student's *t* test and one-way analysis of variance were used.

For *renin* gene knockdown, we transfected renin-siRNAs into HOKs (Figure 4A). Our data suggested that LPS or activated CD4^+^ T cells failed to induce IL-17 expression in the knockdown of *renin* in HOKs (Figures 4B–4E). Importantly, p65-siRNA transfection and Bay 11-7082 treatment both compromised IL-17 increases in OLP cell models (Figures 4F–4I and S4A–S4D), indicating that inhibition of the upstream signaling of renin may regulate IL-17 productions.

### Renin Upregulates IL-17 Expression via Phosphorylating STAT4

**Figure 5:**
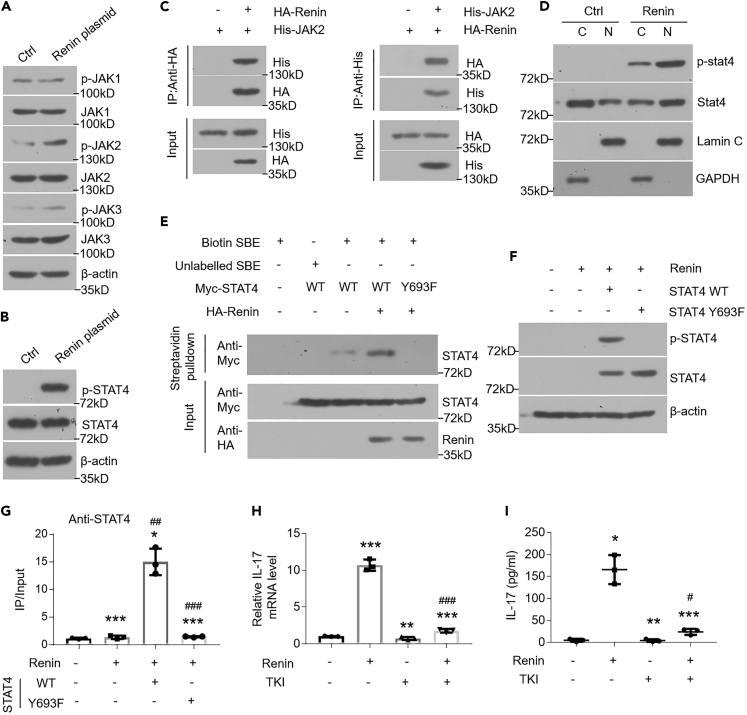
Renin Phosphorylates STAT4 to Induce IL-17 Expression (A) Western blot analysis of HOKs transfected with empty or renin plasmids, using antibodies as indicated. (B) Western blot analysis of STAT4 and phospho-STAT4 in HOKs transfected with empty or renin plasmids. (C) Co-IP and Western blot analyses of cell lysates from HOKs transfected with the indicated plasmids. (D) HOKs were transfected with empty or renin plasmids. Cytoplasmic (C) and nuclear (N) fractions were extracted and analyzed by Western blotting using indicated antibodies. Lamin C is selected to be a nuclear marker, and GAPDH serves as a cytoplasmic marker. (E) Y693F mutation eliminated DNA binding of STAT4 in a pull-down assay. Biotin-labeled SBE probes were added into the transfected HOKs lysates to bind proteins and then pulled down by streptavidin beads. Retrieved proteins were detected by Western blot. Unlabeled SBE probe is chosen for a negative control. (F–I) The endogenous STAT4 in HOKs was deleted by CRISPR/Cas9 system, and different plasmids were transfected into the STAT4-knockout cell line. Western blot was carried out to determine the p-STAT4 and STAT4 levels in transfected HOKs (F). Chromatin immunoprecipitation (ChIP) was performed using anti-STAT4 antibody and showed that Y693F mutation impeded STAT4 binding to the promoter region of *IL-17* gene (G). Real-time PCR (H) or ELISA (I) detection of IL-17 in HOKs transfected with renin plasmids in the presence or absence of TKI. ∗p < 0.05, ∗∗p < 0.01, ∗∗∗p < 0.001 versus corresponding control; #p < 0.05, ##p < 0.01, ###p < 0.001 vs. renin group, n = 3. Ctrl, control; WT, wild type; Y693F, Tyr693 mutant; SBE, STAT4 binding element; TKI, tyrosine kinase inhibitor. Data were shown as means ± SD. Two-tailed Student's *t* test and one-way analysis of variance were used.

In searching for the mechanism by which renin regulates IL-17 productions, we analyzed the human *IL-17* gene promoter and found STAT4 motif (Figure S5A). ChIP data showed that renin plasmid transfection enhanced the binding action between STAT4 and its motif in the *IL-17* promoter region (Figure S5B), implying renin mediates IL-17 transcription through activating STAT4. How does renin influence STAT4 activity? To answer it, we carried out a Co-IP assay using renin and STAT4 plasmids but observed no protein interaction (Figure S5C). The Janus kinase (JAK)/STAT signaling pathway is well known to transduce the activity of cellular signals. Upon binding with signals, JAK proteins are phosphorylated on their tyrosine residues, resulting in recruitment and tyrosine-phosphorylation of STAT proteins (Aaronson and Horvath, 2002, Brooks et al., 2014). To explore this phenomenon, we tested the activities of three types of JAK proteins (JAK1-3) in renin-transfected HOKs. Among these, we found JAK2 to be the only protein phosphorylated by renin (Figure 5A). STAT4 is phosphorylated on tyrosine residue 693 (Tyler et al., 2007). Consistently, STAT4 phosphorylation on Tyr693 was significantly increased in HOKs overexpressing renin (Figure 5B). The roles of renin in JAK2 and STAT4 were also validated using a dose-dependent experiment (Figure S5D). Co-IP assays confirmed the interaction between renin and JAK2 (Figure 5C), uncovering the underpinning whereby renin regulates JAK2 phosphorylation.

We then transfected STAT4 plasmids into HOKs and found STAT4 overexpression has no effect on IL-17 transcription and expression (Figures S5E–S5G), indicating that the overexpression, itself, of STAT4 is not sufficient to induce IL-17 expression and that phosphorylation may be a key event that allows for STAT4's induction of IL-17 expression. To validate it, we confirmed the translocation of phosphorylated STAT4 from cytoplasm into nucleus following renin plasmid transfection (Figure 5D) and detected the increase of phosphorylated STAT4 in the nucleus of oral keratinocytes of diseased biopsies and in the oral epithelial layer of renin-Tg mice (Figures S5H and S5I). In addition, wild-type STAT4 was able to bind efficiently to the biotin-labeled STAT4-binding element (SBE) with renin overexpression, but not Y693F mutant (Figure 5E). Next, we deleted the endogenous *STAT4* gene in HOKs using CRISPR/Cas 9 system (Figure S5J), followed by establishment of cell lines stably expressing wild-type STAT4 or its Y693F mutant (Figure S5K). These results demonstrated that renin phosphorylated Tyr693 on STAT4, which facilitated occupancy of STAT4 on the promoter of *IL-17* in HOKs that express wild-type STAT4 rather than mutant Y693F (Figures 5F and 5G). Tyrosine Kinase Inhibitor (TKI) acting as a tyrosine phosphorylation suppressor blocked renin-induced IL-17 increases (Figures 5H and 5I). Together, these findings support the hypothesis that renin phosphorylates STAT4 on Tyr693 to improve IL-17 transcripts.

### Vitamin D/VDR Signaling Blunts Renin and IL-17 Increases in the OLP Cell Models

**Figure 6:**
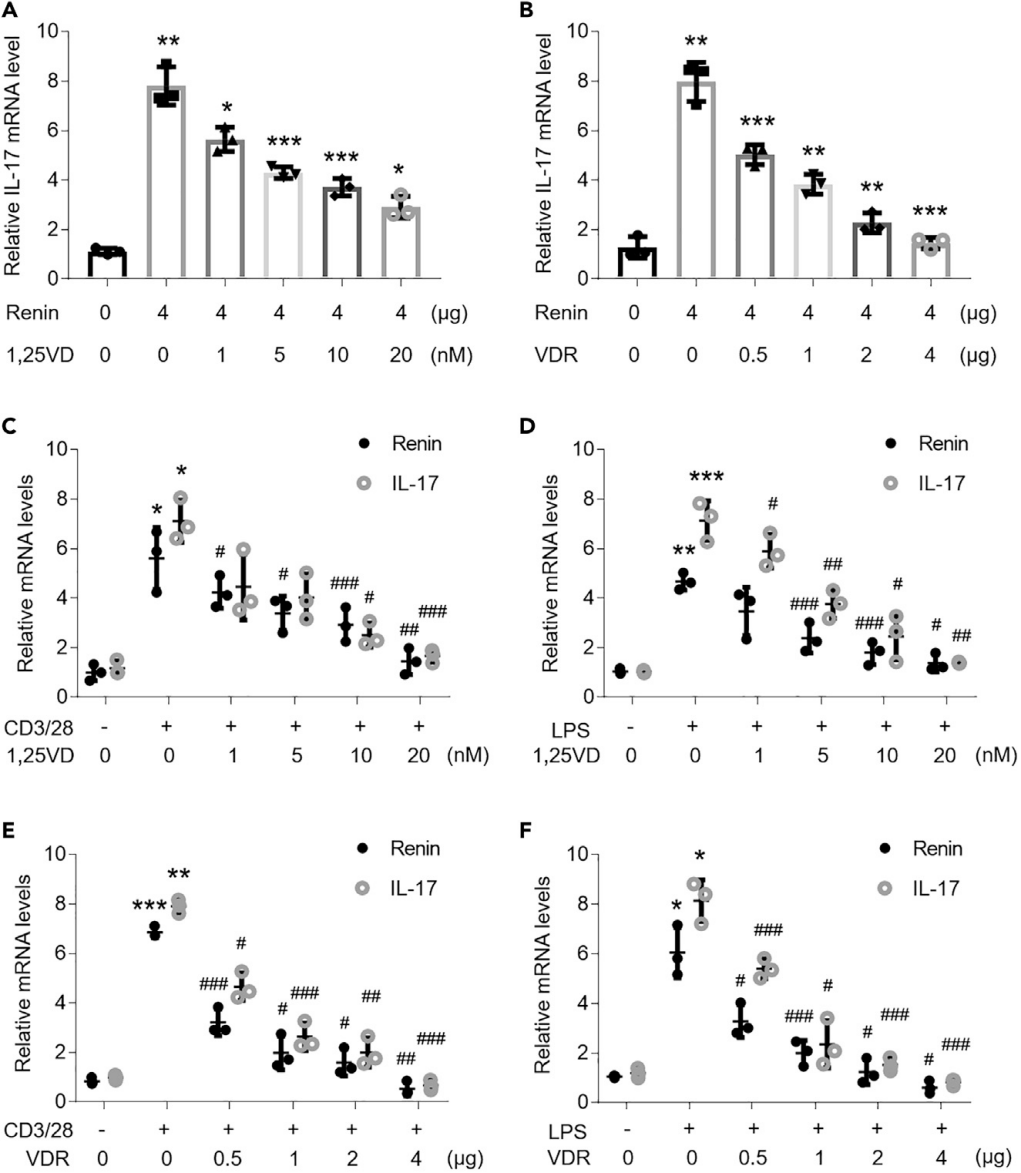
Vitamin D/VDR Signaling Inhibits Renin and IL-17 Transcripts in OLP Cell Models (A) HOKs were pretreated with different doses of 1,25VD for 12 h, and then challenged by renin plasmids transfection for 36 h (B) HOKs were co-transfected with VDR and renin plasmids for 36 h. (C and D) HOKs were pretreated with different doses of 1,25VD for 12 h and then challenged by activated CD4^+^ T cells (C) or 100 ng/mL LPS (D) for 8 h. (E and F) HOKs were transfected with different doses of VDR plasmids for 36 h, followed by 8-h activated CD4^+^ T cells (E) or 100 ng/mL LPS (F) treatment. Renin and IL-17 transcripts were quantified by real-time PCR. ∗p < 0.05, ∗∗p < 0.01, ∗∗∗p < 0.001 versus corresponding control; #p < 0.05, ##p < 0.01, ###p < 0.001 versus LPS or CD3/28 group, n = 3. 1,25VD, 1,25(OH)_2_D_3_. Data were shown as means ± SD. Two-tailed Student's *t* test and one-way analysis of variance were used.

Vitamin D/VDR signaling, which is reported to inhibit cytokine productions and apoptosis of oral keratinocytes, is demonstrated to play a suppressive role in renin expression and RAS (Ge et al., 2019, Li, 2003, Yuan et al., 2007, Zhao et al., 2019). To explain the effects of vitamin D/VDR signaling on renin and IL-17 productions in the context of OLP, we administered different doses of vitamin D and VDR plasmids to HOKs. As shown, both vitamin D and VDR ameliorated renin-induced IL-17 overexpression in HOKs (Figures 6A, 6B, S6A, and S6B). Moreover, the vitamin D-VDR signaling is capable of impeding renin and IL-17 increases in OLP cell models (Figures 6C–6F and S6C–S6J). Together, these data indicate that vitamin D/VDR signaling suppresses renin and IL-17 expression in OLP.

## Discussion

IL-17 has been reported to play potential pathogenic roles in autoimmune disease development, including oral lichen planus (He et al., 2019, Lu et al., 2014). Increased levels of IL-17 have been observed in the biopsies and serum from OLP patients (Lu et al., 2014, Pouralibaba et al., 2013). Some recent studies suggest that renin is robustly elevated in the microenvironment of inflamed tissues, and RAS plays a role in activating Th17 cells and increasing IL-17 production (He et al., 2019, Platten et al., 2009). We examined the roles of renin and IL-17 in OLP and explained the mechanism by which renin upregulates IL-17 in oral keratinocytes.

Renin, an important component of the RAS, has crucial effects on multiple pathophysiological processes (Re, 2004). It is well known that renin exerts its regulatory actions in cardiovascular and renal systems, but the role of local renin in oral keratinocytes remains poorly understood. In this report, we present evidence that renin is dramatically enhanced in the epithelial layer and lamina propria of OLP tissues, a result consistent with other investigations demonstrating renin is induced in colonic inflamed biopsies (He et al., 2019). This finding is confirmed in OLP cell models, where renin is induced by LPS or activated CD4^+^ T cells in a dose-dependent manner. Although previous studies have reported that renin may be secreted from the lymphocytes in the lamina propria, our cell model data suggest that the oral keratinocytes are capable of producing renin in response to stimulation. Bceause there are no well-established animal models for mimicking OLP, animal studies are not involved here.

Previous studies have suggested that cyclic AMP response induces renin production (Yuan et al., 2007), but cyclic AMP signaling is not activated in OLP (Data not shown). Our finding of the κB motif in the promoter of *renin* gene led us to hypothesize that NF-κB pathway may induce renin production in OLP. Consistent with data from our previous studies (Zhao et al., 2018a, Zhao et al., 2018b), we observed that the activated NF-κB pathway in keratinocytes of OLP considerably enhances renin production. Similarly, genetic or chemical interference of NF-κB pathway was associated with decreased renin production. The active NF-κB pathway is described to be induced by proinflammatory cytokines (such as TNF-α) (Chen et al., 2013). Therefore, in the setting of OLP, bacteria or secretions of CD4^+^ T-cell-induced cytokines may activate NF-κB signaling pathway. One limitation is that our results do not consider the possibility that renin may be induced directly by other signaling pathways. However, we explained, at least in part, the cause of upregulated renin in OLP in this study.

We found IL-17 production is dramatically increased in the diseased tissues and serum from patients with OLP, in agreement with other studies (Lu et al., 2014, Pouralibaba et al., 2013). In immune cells, RAS is reported to elevate IL-17 secretions by stimulating mucosal Th17 immunity (He et al., 2019). Inhibition of RAS plays a suppressive role in IL-17 expression in airway diseases (Weber et al., 2012). Similarly, we found renin affects IL-17 productions in the gain-of-function or loss-of-function assays in HOKs. Unlike with other studies (Weber et al., 2012), we did not block RAS with angiotensin II receptor blockers (ARBs) or angiotensin-converting enzyme inhibitors (ACEIs) because we aimed to explore the function of renin in HOKs rather than the whole RAS.

To exploit the mechanism by which renin regulates IL-17, we found SBE in the promoter of *IL-17* gene and confirmed renin takes effect on IL-17 transcripts via STAT4 activation. Furthermore, our data indicate renin interacts with JAK2 to phosphorylate STAT4, which is consistent with the notion that RAS performs its functions in immune cells via JAK2/STAT pathway (He et al., 2019). JAKs phosphorylate STATs on tyrosine residues to transduce cellular signaling (Aaronson and Horvath, 2002). To this end, we assessed STAT4 phosphorylation and found renin phosphorylates tyrosine residue 693 of STAT4 protein. This event helps STAT4 translocate into nucleus to bind to the *IL-17* promoter. It appears that serine residue 721 is another potential site for STAT4 phosphorylation (Morinobu et al., 2002). Therefore, we tested Ser721 phosphorylation by Western blot analysis, wherein we observed overexpression of renin did not lead to the phosphorylation of Ser721 (Data not shown). Moreover, we mutated tyrosine residue 693 and showed STAT4 mutant's regulatory effects on IL-17 transcripts are abolished completely in HOKs with renin plasmids transfection, indicating Tyr693 as the only modification site.

Vitamin D/VDR signaling is proved to suppress both renin and IL-17 levels in OLP in this work, consistent with the point that vitamin D inhibits *renin* gene transcription (Yuan et al., 2007). Because vitamin D/VDR signaling is able to inactivate NF-κB pathway (Chen et al., 2013), they may regulate renin expression via blocking NF-κB pathway in HOKs.

**Figure 7:**
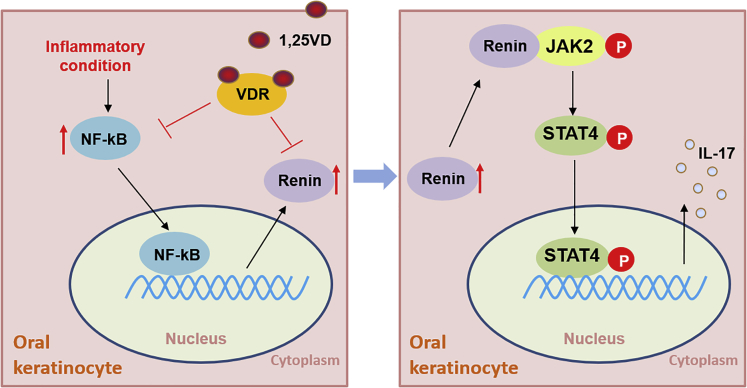
Schematic of Renin's Effects on IL-17 Expression in Oral Keratinocytes

In this report, we present evidence that renin increases STAT4 phosphorylation on Try693 to increased IL-17 productions in keratinocytes of OLP (Figure 7). Some studies suggest that recombinant human IL-17 treatment robustly increases matrix metalloproteinases 9 (MMP9) expression, which results in the deterioration of the mucosal extracellular matrix (Wang et al., 2018). Although the role of IL-17 in apoptosis of oral keratinocytes is still unclear, it is reported to induce apoptosis in hepatocyte (Zhao et al., 2018a, Zhao et al., 2018b), indicating IL-17 may be related with oral keratinocytes apoptosis. Thus, overexpression of renin and IL-17 in OLP may affect the onset and pathogenesis of this disease by damaging the extracellular matrix and inducing apoptosis of oral keratinocytes. Our results provide insights into the understanding of pathogenesis with respect to OLP and possible new therapeutic targets for clinic management of this disease. However, more investigations are required to guarantee the efficiency of this kind of new treatment.

### Limitations of the Study

Because much of the data in this study still rely on overexpression or knockdown in HOK lines, it would be great to perform these experiments in a primary cell system. Moreover, animal models mimicking OLP are required to better explore this kind of disease.

## Methods

All methods can be found in the accompanying Transparent Methods supplemental file.

## Supplemental Information

Document S1. Transparent Methods, Figures S1–S6, and Tables S1 and S2
