# Supplementary material for: Renin Promotes STAT4 Phosphorylation to Induce IL-17 Production in Keratinocytes of Oral Lichen Planus
Source: iScience. 2020 Mar 13;23(4):100983. doi: 10.1016/j.isci.2020.100983 (PMC7093809; doi:10.1016/j.isci.2020.100983)
Supplement: Document S1. Transparent Methods, Figures S1–S6, and Tables S1 and S2 [file mmc1.pdf]

iScience, Volume *23*

Supplemental Information

Renin Promotes STAT4 Phosphorylation to Induce IL-17 Production in Keratinocytes of Oral Lichen Planus

Xuejun Ge, Hanting Xie, Tivoli Nguyen, Bin Zhao, Jing Xu, and Jie Du

Supplemental Figures

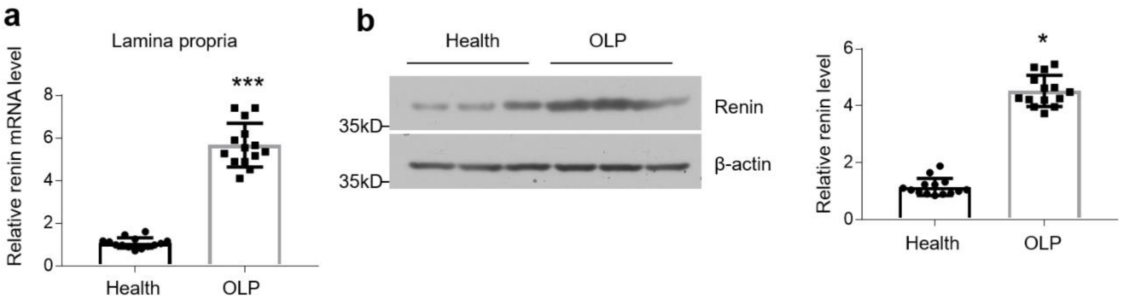

Supplemental figure 1. Renin expression is elevated in the lamina propria of OLP, Related to Figure 1. (a) Real-time PCR quantification of renin in the lamina propria of oral mucosal membranes. (b) Western blot analysis and densitometric quantitation of renin in the lamina propria of oral samples. *P < 0.05, ***P < 0.001 vs. corresponding healthy controls, n=14 each group. Data were shown as means ± SD. 2-tailed Student's *t* test was used.

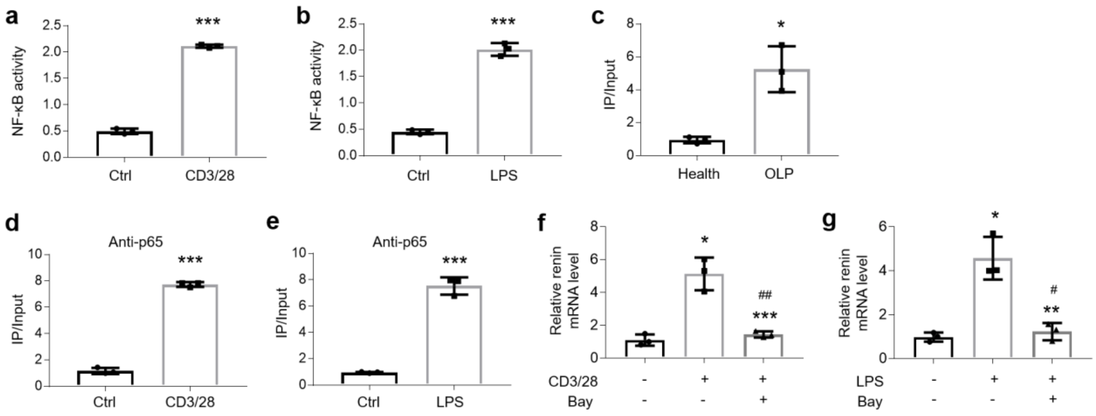

Supplemental figure 2. Effects of NF-κB pathway on renin expression, Related to Figure 2. (a and b) NF-κB activity in HOKs after activated $CD4^+$ T cells (a) or LPS (b) treatment. (c-e) Binding of NF-κB to the promoter of endogenous *renin* was increased in the epithelial layer of OLP biopsies (c), and in HOKs treated with activated $CD4^+$ T cells (d) or 100 ng/ml LPS (e) for 8 hours. Chromatin immunoprecipitation (ChIP) was performed using anti-p65 antibody. (f and g) Real-time PCR quantification of renin mRNA levels in HOKs challenged by activated $CD4^+$ T cells (f) or 100 ng/ml LPS (g) for 8 hours with or without 12-hour Bay 11-7082 (20 nM) pretreatment. *P < 0.05, **P < 0.01, ***P < 0.001 vs. corresponding control; #P < 0.05, ##P < 0.01 vs. LPS or CD3/28 group, n = 3. Ctrl, control; Bay, Bay 11-7082. Data were shown as means ± SD. 2-tailed Student's *t* test and one-way analysis of variance were used.

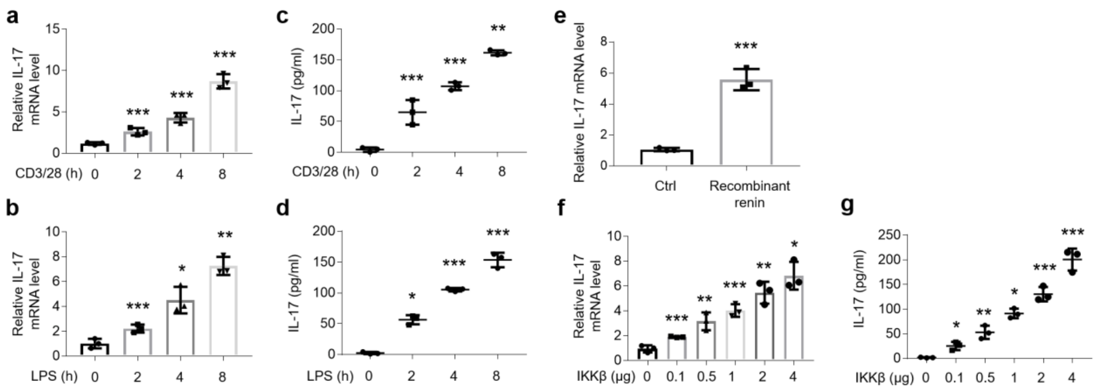

Supplemental figure 3. Expression of IL-17 in OLP or HOKs with IKKβ overexpression, Related to Figure 3. (a and b) Real-time PCR analysis of IL-17 in HOKs with activated $CD4^+$ T cells (a) or 100 ng/ml LPS (b) treatment. (c and d) The amounts of IL-17 productions in culture medium from activated $CD4^+$ T cells- (c) or 100 ng/ml LPS- (d) treated HOKs were assessed by Elisa. (e) Real-time PCR analysis of IL-17 in HOKs treated with or without recombinant renin (50 ng/ml, Enzo Life Sciences, Cat: ENZ-PRT193-0010). (f and g) Real-time PCR (f) or Elisa (g) analysis of IL-17 in HOKs transfected with IKKβ plasmids or culture medium from these cells. n = 3 each group of cell line studies. *P < 0.05, **P < 0.01, ***P < 0.001 vs. corresponding control. Data were shown as means ± SD. 2-tailed Student's *t* test was used.

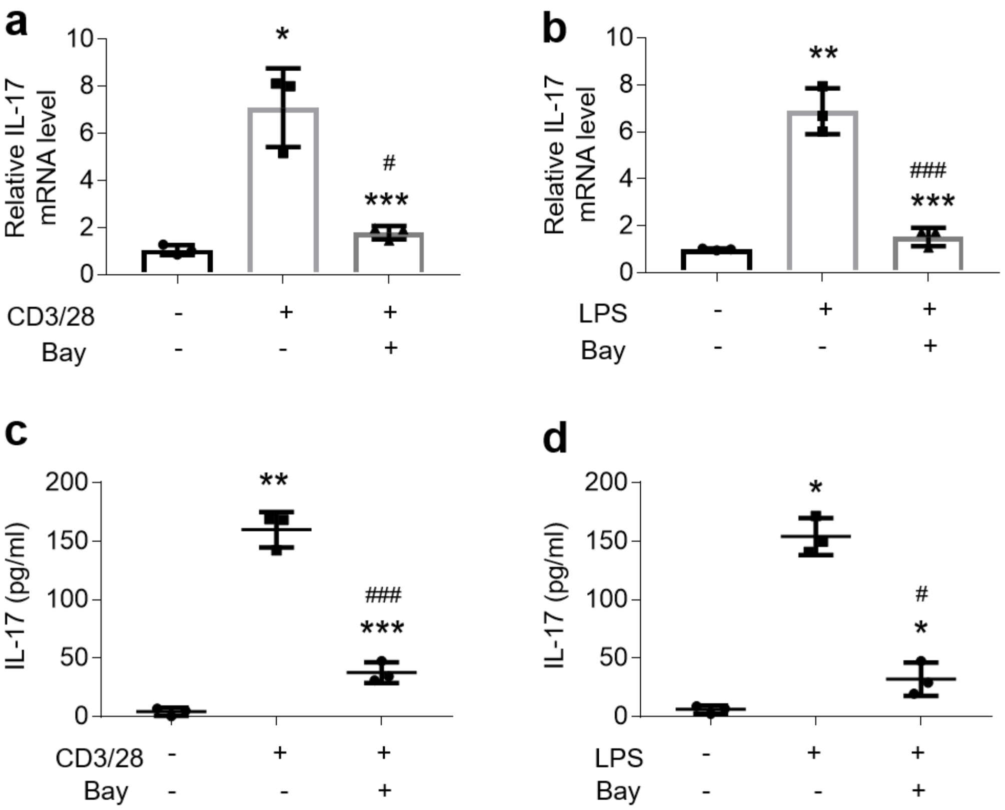

Supplemental figure 4. Inhibition of NF-κB pathway blunts IL-17 increases in OLP cell models, Related to Figure 4. (a and b) Real-time PCR quantification of IL-17 mRNA levels in HOKs treated with activated $CD4^+$ T cells (a) or 100 ng/ml LPS (b) for 8 hours, with or without 12-hour Bay compound pretreatment. (c and d) Elisa assessment of IL-17 secretions in the culture medium from HOKs stimulated with activated $CD4^+$ T cells (c) or 100 ng/ml LPS (d) for 8 hours, following 12-hour Bay compound pretreatment. *P < 0.05, **P < 0.01, ***P < 0.001 vs. corresponding control; #P < 0.05, ###P < 0.001 vs. LPS or CD3/28 group, n = 3. Data were shown as means ± SD. 2-tailed Student's *t* test and one-way analysis of variance were used.

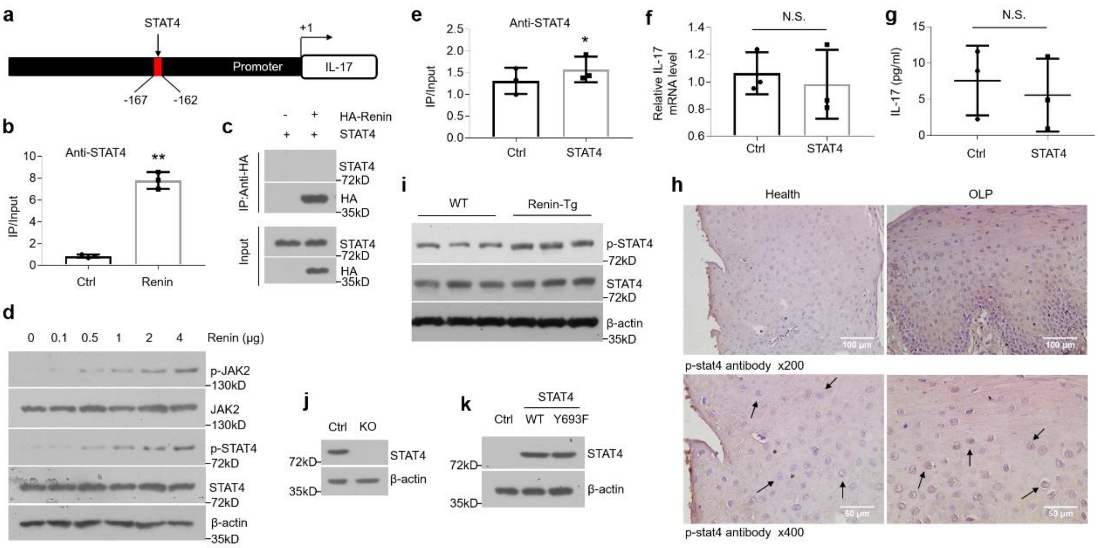

Supplemental figure 5. The occupancy of STAT4 on the promoter of *IL-17* gene, Related to Figure 5. (a) Schematic illustration of STAT4 motif in the promoter of *Il-17* gene. (b) Renin plasmids transfection in HOKs helps STAT4 bind to the promoter of endogenous *IL-17*. Chromatin immunoprecipitation was performed using anti-STAT4 antibody. (c) Co-IP and western blot analyses of cell lysates from HOKs transfected with the indicated plasmids. (d) Western blot analysis of proteins in renin-transfected HOKs lysates. (e) Chromatin immunoprecipitation analysis of empty or STAT4 plasmids-transfected HOKs using anti-STAT4 antibody. (f and g) Real-time PCR (f) and Elisa (g) measurements of IL-17 expression in HOKs transfected with empty or STAT4 plasmids. (h) phospho-STAT4 immunostaining in the oral tissues of healthy controls and OLP patients. (i) Western blot analysis of proteins in mice oral epitheliums lysates. (j) Endogenous STAT4 in HOKs was deleted using CRISPR/Cas9 system and demonstrated by western blot. (k) Wild-type STAT4 and Y693F mutant were transfected into HOKs in which the endogenous STAT4 was knocked out. The expression of STAT4 was detected by western blot. n = 5 each group of mice samples, n = 3 each group of cell line studies. *P < 0.05, **P < 0.01 vs. corresponding control. Ctrl, control. Data were shown as means ± SD. 2-tailed Student's *t* test was used.

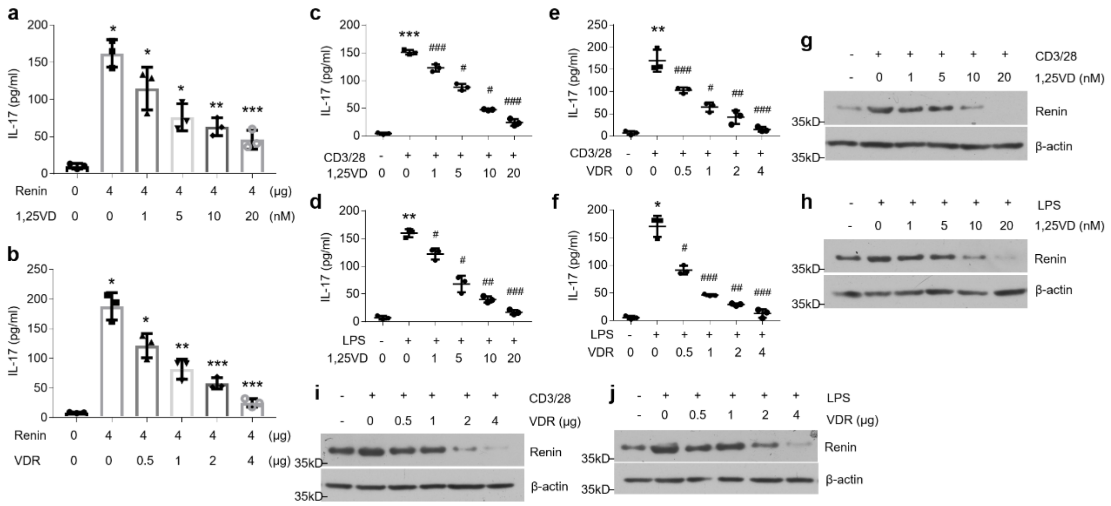

Supplemental figure 6. Vitamin D/VDR signaling inhibits renin and IL-17 protein expression in OLP cell models, Related to Figure 6. (a) Elisa test of culture medium of HOKs which were pretreated with different doses of 1,25VD for 12 hours, and then challenged by renin plasmids transfection for 36 hours. (b) Elisa detection of culture medium of HOKs which were co-transfected with VDR and renin plasmids for 36 hours. (c-j) HOKs were pretreated with different doses of 1,25VD (12 hours) or of VDR plasmids (36 hours), and then challenged by activated $CD4^+$ T cells or 100 ng/ml LPS. Real-time PCR quantification of IL-17 in HOKs (c and d), Elisa detection of IL-17 secretions in the culture medium (e and f), western blot analysis of renin (g and j). *P < 0.05, **P < 0.01, ***P < 0.001 vs. corresponding control; #P < 0.05, ##P < 0.01, ###P < 0.001 vs. LPS or CD3/28 group, n = 3. 1,25VD, $1,25(OH)_2D_3$. Data were shown as means ± SD. 2-tailed Student's *t* test and one-way analysis of variance were used.

Supplemental Tables

Supplemental table 1. Primer sequences involved in this work, Related to Figure 1 and Figure 5.

Primer name	Forward(5'-3')	Reverse(5'-3')
hRenin	ACCTTTGGTCTCCCGACAGA	CACCTCGTTCCTTCAGGCTTT
hIL-17	TCCCACGAAATCCAGGATGC	GGATGTTCAGGTTGACCATCAC
hGADPH	ACCACAGTCCATGCCATCAC	TCCACCACCCTGTTGCTGTA
mIL-17	TCCCTCTGTGATCTGGGAAG	AGCATCTTCTCGACCCTGAA
mGAPDH	TGTGTCCGTCGTGGATCTGA	CCTGCTTCACCACCTTCTTGA
Renin (ChIP)	GGTAATAAATCAGGGCAGAGCA	GGTAATAAATCAGGGCAGAGCA
IL-17 (ChIP)	TGCCCTTCCCATTTTCCTTCA	TGGTCACTTACGTGGCGTG

Supplemental table 2. Antibodies information, Related to Figure 1-5.

Antibody	Source	Identifier
Anti-Renin	Santa Cruz	sc-27318
Anti-β-actin	Santa Cruz	sc-47778
Anti-NF-κB p65	Cell Signaling	8242
Anti-Phospho-NF-κB p65	Cell Signaling	3031
Anti-IKKβ	Cell Signaling	8943
Anti-p-JAK1	Invitrogen	PA5-37617
Anti-JAK1	BD Bioscience	610231
Anti-p-JAK2	Cell Signaling	3771
Anti-JAK2	Cell Signaling	3230
Anti-p-JAK3	Cell Signaling	5031
Anti-JAK3	Cell Signaling	8863
Anti-Phospho-STAT4 (Tyr693)	Cell Signaling	5267
Anti-STAT4	Cell Signaling	2653
Anti-HA	Cell Signaling	3724
Anti-His	Cell Signaling	12698
Anti-Myc	Cell Signaling	2276
Anti-Lamin C	Abcam	Ab125679
Anti-GAPDH	Sigma	G8795
Anti-IL-17	Abcam	ab79056

Transparent Methods

Human samples collection. Human oral mucosal tissues and blood samples were collected from participants at the Stomatological Hospital of Shanxi Medical University. Unaffected control samples were got from volunteers who underwent wisdom teeth extraction. Modified World Health Organization (WHO) diagnostic criteria were used for selecting for OLP patients. The human sample studies were approved by the Institutional Ethical Committee of Shanxi Medical University. Written informed consent was got from each participant involved in this investigation. Detailed information regarding OLP patients was in detail elaborated here (Zhao et al., 2019). There were 6 males and 8 females in OLP patients. Most of patients were middle-aged and all of them showed the reticular subtype.

Cell culture. Human oral keratinocytes (HOKs, ScienCell, Catalog #2610) were cultured in oral keratinocyte medium (ScienCell, Catalog #2611) with 10% FBS. There are two methods for mimicking OLP *in vitro*. In one, HOKs were challenged with 100 ng/ml lipopolysaccharide (LPS, Sigma-Aldrich, O111:B4 *E. coli*) for 8 hours. In the other, HOKs were treated for 8 hours with the culture medium from $CD4^+$ T cells, which were activated with or without anti-CD3/CD28. The supernatant of $CD4^+$ T cell cultures was added to HOKs at a 30% final volumetric concentration (Zhao et al., 2019). In another assay, HOKs were treated with 1,25 VD or Bay 11-7082 compound for 12 hours or transfected with plasmids or siRNAs for 36 hours prior to 8-hour LPS or activated $CD4^+$ T cells challenge.

Isolation and stimulation of $CD4^+$ T cells. Peripheral blood from OLP patients was subjected to Ficoll-Hypaque density gradient centrifugation. Anti-CD4 magnetic particles (BD Biosciences) were used to purify $CD4^+$ T cells. After suspension in RPMI 1640 medium, $CD4^+$ T cells were stimulated by anti-CD3 and anti-CD28 antibodies (BD Biosciences Pharmingen).

Animal studies. Transgenic mouse line RenTgMK 108 (RenTg) used in this study carries a single copy of mouse renin transgene which is driven by liver-specific albumin 109 promoter/enhancer (He et al., 2019). 8-week-old mice were chosen for

this study, they were sex matched and cohoused. All animal studies were approved by the Institutional Ethical Committee affiliated with Shanxi Medical University.

Oral mucosal epithelium isolation. Oral epithelial layer separation assays were performed according to ZB et al (Zhao et al., 2019). Briefly, whole oral buccal tissues from individuals or mice were digested for 12 hours by 0.25% dispase II, followed by direct separation with muscle forceps. After epithelial layer separation, the rest of tissues are lamina propria.

CRISPR/Cas9-regulated knockout of *STAT4.* sgRNA sequence targeting the *STAT4* gene (5'- TGACACGTTGTACCAAATGA-3') was inserted into lentiCRISPRv2 vector (Addgene, catalog 52961) by the BsmBI restriction enzyme. Lenti-vector and packaging plasmids (pMD2.G and psPAX2) were cotransfected into HEK293T cells. Virus was isolated from the culture medium of HEK293T cells after 48-hour transfection. Lentivirus particles were added into HOKs with polybrenes (4 μg/ml) supplement. Puromycin (0.5 μg/ml) was used to select transduced cells.

RT-PCR. Total RNAs were isolated from cells or tissues with TRIzol reagent (Invitrogen). mRNAs were converted to first-stranded cDNAs by a PrimeScript reagent kit (TaKaRa). Real-time PCR was performed with a SYBR Premix kit (TaKaRa). GAPDH was chosen as an internal control. Relative quantification of transcripts was analyzed using the $2^{-\Delta\Delta Ct}$ formula. Primers sequences were provided in supplemental table 1.

Western blot. Western blot analyses were carried out according to previous studies(Zhao et al., 2019). Briefly, cells were dissolved into lysis buffer containing protease inhibitors (Roche) and heated at 95°C for 5 min. The same amount of protein was separated by SDS-PAGE and electrotransferred onto PVDF membranes (Millipore). The membranes were incubated at cold room overnight using primary antibodies, following one-hour blocking buffer treatment. On day 2, HRP-conjugated secondary antibodies were selected to incubate membranes at room temperature for 1 hour. Bands were detected by ECL kit (Thermo Fisher Scientific) and visualized with x-ray films at dark room. More details on primary antibodies are provided in supplemental table 2.

Immunohistochemical staining. Immunohistochemical assays were conducted according to ZB et al (Zhao et al., 2019). Oral tissues were fixed in 10% formalin and embedded with paraffin. Blocks were cut into 4 μm slides using a microtome (Leica). After deparaffinization and dehydration, slides were treated with citrate buffer for antigen retrieval and hydrogen peroxide for endogenous peroxidase activity blockage. Primary antibodies (anti-Renin, anti-p-STAT4 and anti-IL-17) were used to incubate slides at cold room overnight, followed by one-hour secondary incubation. After a series of washes, antibody-connected tissues were treated with 3,3′-Diaminobenzidine (DAB) and hematoxylin. Slides were observed under a light microscope. More details on primary antibodies are provided in supplemental table 2.

Chromatin immunoprecipitation (ChIP). Cells were washed with PBS for 3 times, followed by 10-min crosslinking with 1% formaldehyde. ChIP assays were carried out using a commercial kit (Pierce) according to the manufacturer's instructions. Anti-p65 and anti-STAT4 antibodies were involved in this assay.

DNA pulldown assay. DNA pulldown assay was performed according to ZQ et al (Zhang et al., 2019). Briefly, HOKs transfected with plasmids were lysed in a DNA binding buffer, followed by 30-min incubation with 30 pmol biotinylated STAT4-binding element (SBE) oligonucleotides (CATTGGGGGCGGAAATTTTAACCAA). DNA-protein complexes were precipitated via streptavidin beads, and then subjected to western blot analysis.

Co-immunoprecipitation (Co-IP). Co-IP assays were performed according to ZG et al (Zhao et al., 2019). Briefly, transfected cells were lysed and supernatants were collected before incubation with antibodies and protein A/G beads (Thermo Fisher Scientific). After 4-hour incubation, complexes were washed with NETN buffer 4 times and then subjected to SDS-PAGE for analyses.

Elisa. Blood samples obtained from participants were stored at cold room overnight for serum collection. IL-17 levels in human serum samples, HOKs culture medium and the lysates of mice oral epithelial layer were detected using a commercial Elisa kit (eBioscience) according to the instructions. The OD values were monitored by a microplate reader.

Transfection assay. Plasmids and siRNAs were transfected into HOKs with Lipofectamin 3000 (Invitrogen). HA-Renin plasmids were obtained from Sino Biological (Cat: HG10969-CY), His-JAK2 plasmids from Biological (Cat: HG11198-CH), and Myc-STAT4 plasmids from Origene (Cat: RC206892). Y693F mutant of STAT4 was performed with the Site-Directed Mutagenesis Kit (Clontech). Oligonucleotides (5'-GACAAGGGTTTCGTCCCTTCTGTTTTTATCCC-3') were used to change tyrosine to phenylalanine at codon 693 (Morinobu et al., 2002; Visconti et al., 2000). Renin-siRNAs (Cat: sc-41644) and NF-κB p65-siRNAs (sc-29410) were got from Santa Cruz.

NF-κB activity assay. NF-κB activity assay was carried out by using NF-κB luciferase reporter kit (BPS Biosci-ence). Primary oral keratinocytes from human samples were isolated and cultured in Petri dishes. Both HOKs and primary oral keratinocytes were transfected with NF-κB luciferase reporter using Lipofectamine 3000. The luciferase activity was detected by Dual Luciferase Assay System.

Statistical analysis. Data were shown as means ± SD. Statistical analyses were performed by 2-tailed Student's *t* test or one-way analysis of variance for 2 or multiple groups, respectively. $P < 0.05$ was considered to be statistically significant.

Supplemental References

He, L., Du, J., Chen, Y., Liu, C., Zhou, M., Adhikari, S., Rubin, D.T., Pekow, J., and Li, Y.C. (2019). Renin-angiotensin system promotes colonic inflammation by inducing TH17 activation via JAK2/STAT pathway. Am J Physiol Gastrointest Liver Physiol *316*, 774-784.

Morinobu, A., Gadina, M., Strober, W., Visconti, R., Fornace, A., Montagna, C., Feldman, G.M., Nishikomori, R., and O'Shea, J.J. (2002). STAT4 serine phosphorylation is critical for IL-12-induced IFN-gamma production but not for cell proliferation. P Natl Acad Sci USA *99*, 12281-12286.

Visconti, R., Gadina, M., Chiariello, M., Chen, E.H., Stancato, L.F., Gutkind, J.S., and O'Shea, J.J. (2000). Importance of the MKK6/p38 pathway for interleukin-12-induced STAT4 serine phosphorylation and transcriptional activity. Blood *96*, 1844-1852.

Zhang, Q., Xiao, M., Gu, S., Xu, Y., Liu, T., Li, H., Yu, Y., Qin, L., Zhu, Y., Chen, F.*, et al.* (2019). ALK phosphorylates SMAD4 on tyrosine to disable TGF-beta tumour suppressor functions. Nat Cell Biol *21*, 179-189.

Zhao, B., Xu, N., Li, R., Yu, F.Y., Zhang, F., Yang, F., Ge, X.J., Li, Y.C., and Du, J. (2019). Vitamin D/VDR signaling suppresses microRNA-802-induced apoptosis of keratinocytes in oral lichen planus. Faseb Journal *33*, 1042-1050.

Zhao, G., Gong, L., Su, D., Jin, Y., Guo, C., Yue, M., Yao, S., Qin, Z., Ye, Y., Tang, Y.*, et al.* (2019).

Cullin5 deficiency promotes small-cell lung cancer metastasis by stabilizing integrin beta1. J Clin Invest *129*, 972-987.
